# Individual karyotypes at the origins of cervical carcinomas

**DOI:** 10.1186/1755-8166-6-44

**Published:** 2013-10-17

**Authors:** Amanda McCormack, Jiang Lan Fan, Max Duesberg, Mathew Bloomfield, Christian Fiala, Peter Duesberg

**Affiliations:** 1 Department of Molecular and Cell Biology; Donner Laboratory, University of California at Berkeley, Berkeley, CA, USA; 2 Gynmed Ambulatorium, Mariahilferguertel 37, 1150, Vienna, Austria

**Keywords:** Karyotype arrays, Speciation theory of cancer, Individuality of cancers, Clonality of cancers, Intrinsic and acquired drug-resistance, 1-to-1 chromosome-transcriptome balances, Chromosome recombination index

## Abstract

**Background:**

In 1952 Papanicolaou et al. first diagnosed and graded cervical carcinomas based on individual “abnormal DNA contents” and cellular phenotypes. Surprisingly current papilloma virus and mutation theories of carcinomas do not mention these individualities. The viral theory holds that randomly integrated, defective genomes of papilloma viruses, which are often untranscribed, cause cervical carcinomas with unknown cofactors 20–50 years after infection. Virus-free carcinomas are attributed to mutations of a few tumor-suppressor genes, especially the p53 gene. But the paradox of how a few mutations or latent defective viral DNAs would generate carcinomas with endless individual DNA contents, degrees of malignancies and cellular phenotypes is unsolved. Since speciation predicts individuality, we test here the theory that cancers are autonomous species with individual clonal karyotypes and phenotypes. This theory postulates that carcinogens induce aneuploidy. By unbalancing mitosis genes aneuploidy catalyzes chain reactions of karyotypic evolutions. Most such evolutions end with non-viable karyotypes but a few become new cancer karyotypes. Despite congenitally unbalanced mitosis genes cancer karyotypes are stabilized by clonal selections for cancer-specific autonomy.

**Results:**

To test the prediction of the speciation theory that individual carcinomas have individual clonal karyotypes and phenotypes, we have analyzed here the phenotypes and karyotypes of nine cervical carcinomas. Seven of these contained papilloma virus sequences and two did not. We determined phenotypic individuality and clonality based on the morphology and sociology of carcinoma cells in vitro. Karyotypic individuality and clonality were determined by comparing all chromosomes of 20 karyotypes of carcinomas in three-dimensional arrays. Such arrays list chromosome numbers on the x-axis, chromosome copy numbers on the y-axis and the number of karyotypes arrayed on the z-axis. We found (1) individual clonal karyotypes and phenotypes in all nine carcinomas, but no virus-specific markers, (2) 1-to-1 variations between carcinoma-specific karyotypes and phenotypes, e.g. drug-resistance and cell morphology, (3) proportionality between the copy numbers of chromosomes and the copy numbers of hundreds of over- and under-expressed mRNAs, (4) evidence that tobacco-carcinogens induce cervical carcinomas via aneuploidy, consistent with the speciation theory.

**Conclusions:**

Since the individual clonal karyotypes of nine carcinomas correlated and co-varied 1-to-1 with complex individual transcriptomes and phenotypes, we have classical genetic and functional transcriptomic evidence to conclude that these karyotypes encode carcinomas - much like the clonal karyotypes that encode conventional species. These individual karyotypes explain the individual “DNA contents”, the endless grades of malignancies and the complex individual transcriptomes and phenotypes of carcinomas.

## Background

In 1952 Papanicolaou et al. first diagnosed and graded cervical carcinomas microscopically based on individual “abnormal DNA contents” and cellular phenotypes [1]. These individualities of cervical carcinomas have since been confirmed and extended by numerous cytometric and cytogenetic studies [2-21]. Based on these classic characteristics ‘Pap-tests’ are used to this day to diagnose and grade carcinomas.

Surprisingly, the current papilloma virus and mutation theories of carcinomas do not mention the individual DNA contents and phenotypes of carcinomas. The viral theory holds that randomly integrated defective viral genomes, which are non-immunogenic and often untranscribed, cause cervical carcinomas with unknown cofactors 20–50 years after infection [13,17,18,21-36]. Since defective and inactive papilloma viral sequences are present in at least 50 million (30%) of cancer-free American females [37] as compared to 12,000 annual cervical carcinomas [38], these cofactors must be very rare events. On the other hand the virus-free carcinomas are attributed to mutations of certain tumor-suppressor genes, especially the p53 gene [12,13,16,39-45]. But the paradox of how either a few mutations or latent defective viral genomes generate carcinomas with endless “abnormal DNA contents”, grades of malignancies and phenotypic individualities is unsolved [1].

**Figure 1:**
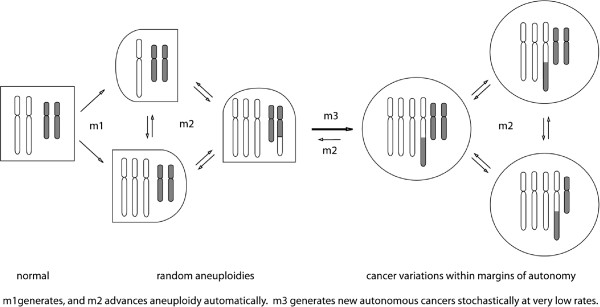
**The speciation theory of cancer: Carcinogenesis in one stochastic karyotypic step.** The speciation theory postulates that carcinogens or spontaneous accidents induce aneuploidy, i.e., losses or gains of chromosomes at dose-dependent rates, termed m1. By unbalancing chromosomes and thus mitosis genes aneuploidy initiates chain reactions of automatic karyotypic evolutions at aneuploidy-dependent rates, termed m2. Most such evolutions end with non-viable karyotypes, but a few become new cancer karyotypes at very low rates, termed m3 – in one stochastic step, as non-autonomous precursors are too unstable to accumulate. Because of its congenital “aneuploidy” (relative to normal) the cancer karyotype is variable at m2-rates, but is stabilized by clonal selection for autonomy within cancer-specific margins. Since aneuploidy changes the normal phenotype (squares), aneuploid cells are shown as half round-half squares and cancer cells are shown as circles.

Since speciation predicts individuality, we test here the theory that cancers are autonomous species with new individual karyotypes and phenotypes [46-53]. This theory postulates that carcinogens or spontaneous events initiate speciation by inducing aneuploidy. Aneuploidy catalyzes chain reactions of karyotypic evolutions automatically, because it unbalances chromosomes and thus mitosis genes [54-58]. Most such evolutions end with non-viable karyotypes, but a few become new cancer karyotypes at very low rates ‘one cancer-one karyotype’ [50,57,58] (Figure 1). Despite congenitally unbalanced mitosis genes cancer karyotypes are stabilized [46,50,59-62], indeed immortalized [58] by clonal selections for cancer-specific autonomy (Figure 1). The resulting karyotypic flexibility and corresponding clonal heterogeneity are thus proportional to the degrees of cancer-specific aneuploidy [55,59,60,62,63] (see below).

The relevance of this theory to cervical carcinomas is supported by: (1) A plethora of classic evidence for abnormal DNA contents or aneuploidies in cervical carcinomas [1-21,64-67], (2) Recent evidence that the copy numbers of the mRNAs of individual carcinomas [33,45,68-72], like those of other cancers [73-78], are proportional to the copy numbers of the corresponding carcinoma-specific chromosomes (See Results regarding transcriptomes of cervical carcinomas), and (3) Induction of cervical carcinomas by aneuploidogenic cigarette smoke-carcinogens as predicted by the speciation theory [27,79-83].

In this study we have investigated the predictions of the speciation theory that, (1) Cervical carcinomas have individual clonal karyotypes, transcriptomes and phenotypes irrespective of integrated viral sequences, (2) There are 1-to-1 correlations between phenotypic and karyotypic variations, e.g. intrinsic and acquired drug-resistance and cell morphology, (3) The copy numbers of hundreds of over- and under-expressed mRNAs of carcinomas are proportional to the copy numbers of the corresponding chromosomes of individual carcinomas, (4) Cigarette smoke carcinogens can initiate carcinomagenesis by inducing aneuploidy. As we show below our investigations have indeed confirmed all of these predictions of the speciation theory.

## Results

### Individual phenotypes of cervical carcinomas

The speciation theory predicts that cervical carcinomas have individual clonal phenotypes based on individual clonal karyotypes (Background). To test this prediction we analyzed the phenotypes and karyotypes of nine individual cervical carcinomas. Relevant cytogenetic and phenotypic data of these nine carcinomas are summarized in Table 1. As is shown in this table and in the literature, two of the nine carcinomas, namely C-4II and C-33A are near-diploid, and seven, namely SiHa, HT-3, ME-180, MS-751, CaSki, SW-756 and HeLa are near-triploid [14,18,19,21,40,42,43,84]. Both C-33A and HT-3 are papilloma virus-free, indicating that these carcinomas are independent of papilloma viruses, like other cervical carcinomas not studied here [12,16,40,43-45,67,84]. As per Table 1 and the literature, the remaining seven carcinomas studied here are positive for various DNA sequences from various papilloma virus strains and are thus potentially virus-dependent [14,16-19,21,29,40,43,71].

**Table 1:** Karyotypic and phenotypic individualities of the nine cervical carcinomas of this study

**Carcinoma**	HPV*	**Cell morphology and sociology**	**Ploidy (Avg. Chromosome no)**
C-33A	–	Cobblestone shapes; tight 3D colonies	85% Near-diploid (46±1)
			15% Near-tetraploid (87±2)
HT-3	–	Polymorphic; 3D	80% Near-triploid (68±3)
			20% Near-hexaploid (133±1)
C-4II	+	Polygonal; growing as dense monolayers	Near-diploid (43±1)
CaSki	+	Polymorphic; 3D	Near-triploid (75±3)
HeLa-R	+	Round; tight 3D colony	Near-triploid (71±3)
HeLa-S	+	Fusiform; tight 3D colony	Near-triploid (67±2)
ME-180	+	Triangular; high density	Near-triploid (63±2)
MS-751	+	Polygonal; refractile; tight 3D colonies	Near-triploid (75±10)
SiHa	+	Round or oval; dense gradually growing 3D colonies	Near-triploid (66±4)
SW-756-C1	+	Round	Near-triploid (80±3)
SW-756-C2	+	Fusiform	Near-triploid (82±2)

*HPV = Human papilloma virus; 3D = three dimensional.

#### ***Individual cell morphologies and sociologies***

**Figure 2:**
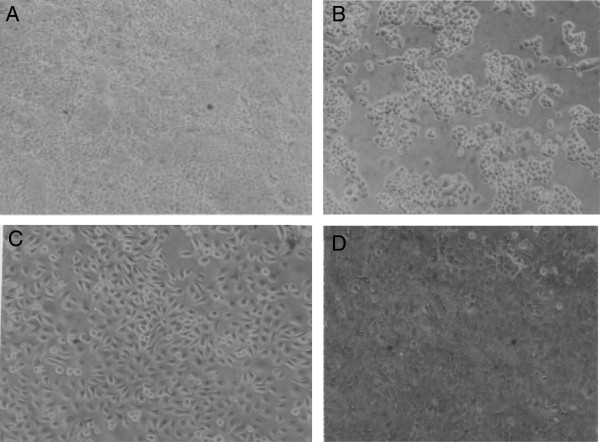
**Cellular morphologies of cervical carcinomas C-4II, C-33A, ME-180 and CaSki.** The cells were grown in medium containing 5% fetal calf serum and photographed at 120× magnification in cell culture dishes (Methods). The following individualities were observed: **(A)** Polygonal C-4II cells forming a dense monolayer, **(B)** Cobblestone shaped and refractile of C-33A cells forming three-dimensional colonies, **(C)** Oval or triangular ME-180 cells forming a dense refractile monolayer, and **(D)** Polymorphic CaSki cells forming a three-dimensional cell layer.

The cellular morphology and sociology of the nine carcinomas was studied microscopically on cells growing under identical conditions in plastic culture dishes (Table 1). Figure 2A shows that under our conditions the cells of the near-diploid C-4II carcinoma had polygonal morphology and grew as relatively flat, dense monolayers. Under the same conditions the cells of near-diploid C-33A carcinoma had cobblestone shapes and grew as tight three-dimensional colonies (Figure 2B). The near-triploid ME-180 cells were triangular and grew to high densities (Figure 2C), and the near-triploid CaSki cells were polymorphic and also grew three-dimensionally (Figure 2D).

**Figure 3:**
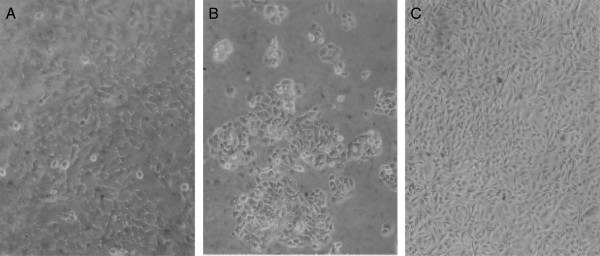
**Cellular morphologies of cervical carcinomas HT-3, MS-751 and CaSki.** The micrographs were prepared as described for Figure 2. The following individualities were observed: **(A)** Polymorphic HT-3 cells forming a near-three-dimensional cell layer, **(B)** Oval and refractile MS-751 cells forming tight, three-dimensional colonies, and **(C)** Round to oval, refractile SiHa cells forming a very dense cell layer.

Furthermore, we observed that the near-triploid HT-3 carcinoma cells were polymorphic and grew three-dimensionally (Figure 3A), and that the MS-751 carcinoma cells were polygonal and refractile and grew as tight three-dimensional colonies (Figure 3B). At variance, SiHa carcinoma cells were round or oval-shaped and grew to very high, gradually increasing three-dimensional densities (Figure 3C).

**Figure 4:**
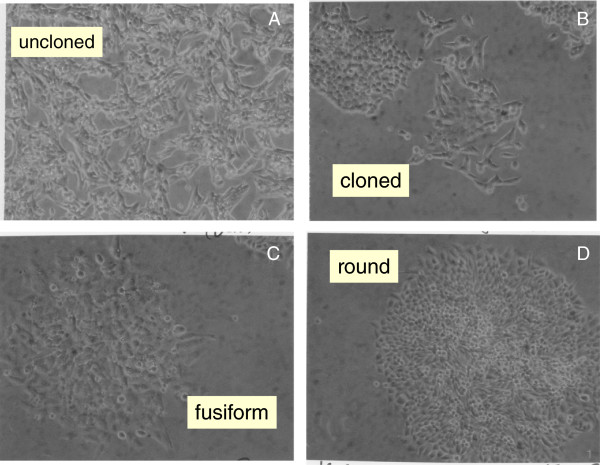
**Cellular morphologies of two morphological variants of the cervical carcinomas SW-756 and of HeLa.** The micrographs were prepared as described for Figure 2. The following individual cell morphologies and sociologies were observed: **(A)** Morphologically heterogeneous SW-756 cells forming a mixed population of fusiform and round cell variants, **(B)** Clonal colonies of fusiform and round SW-756 variants, **(C)** A focus of fusiform HeLa cells, **(D)** A focus of round HeLa cells.

Unexpectedly, in view of long passage history of the SW-756 and HeLa carcinoma lines, we found that both lines were morphologically heterogeneous. The morphological pattern of native SW-756 consisted of fusiform and round cell variants forming the dense three-dimensional cell layers shown in Figure 4A. Micrographs of a fusiform and a round SW-756 variant cloned from the native carcinoma are shown in Figure 4B. Likewise we cloned a fusiform and a round morphological cell variant from a commercial stock of the HeLa carcinoma line. Figures 4C,D show three-dimensional foci of the fusiform and the round cell variant of the HeLa line, respectively. The two morphological HeLa variants isolated by us here are probably the same as those originally cloned and karyotyped by Marguerite Vogt in 1959 [85]. Thus both of these carcinomas consist of two morphologically distinct types of cells, which must be equally stable considering the long passage history of these lines.

It is shown below that the morphological variants of SW-756 and of HeLa are karyotypically closely related (see, *Do karyotypic variations or mutations alter the morphology and drug-sensitivity of carcinomas?*). This suggests that cancer-specific karyotypic variations, rather than mutations would have generated these morphological variants.

#### ***Individual intrinsic drug-resistances***

The cervical carcinomas studied here also differed from each other in intrinsic resistances to cytotoxic drugs. According to the speciation theory this characteristic of cancers is a direct consequence of the karyotypic origins of cancers as follows: Since specific combinations of multi-genic chromosomes generate new autonomous cancer species, numerous new gene combinations with new phenotypes are inevitably generated non-selectively, which are not necessary for carcinogenesis – such as intrinsic drug-resistance [86-88]. Intrinsic drug-resistance is thus a drug treatment-independent, individual phenotype of cancers.

Among the nine carcinoma lines tested here all but one were found to be sensitive to methotrexate at 1 μg per 10^6 cells, while SiHa appeared to be partially resistant (Table 2). In contrast five, namely HT-3, CaSki MS-751, SiHa, ME-180 were highly resistant and the remaining four were modestly resistant to cytosine arabinoside at 1 μg per 10^6 cells. Further, four carcinomas, namely CaSki, MS-751, SW-756, and ME-180 were resistant to puromycin at 1 μg per 10^6 cells (Table 2). These individual intrinsic drug resistances of the nine carcinomas are thus compatible with the prediction of non-essential new cancer phenotypes by the speciation theory.

**Table 2:** Intrinsic drug‒resistances of cervical carcinoma lines

**Carcinoma-lines**	Methotrexate*	Arabinocytidine*	Puromycin*
C-33	-	-/+	-
HT-3	-	+	-
CaSki	-	+	+
MS-751	-	+	+
SW-56	-	-/+	+
C-4II	-	-/+	-
HeLa	-	-/+	-
SiHa	+/-	+	+/-
ME-180	-	+	+/-

*at 1 μg per 10^6 cells; + = confluent culture.

*Section-specific conclusions.* We conclude that all nine distinct cervical carcinomas studied here have individual cell morphologies and individual intrinsic resistances against cytotoxic drugs. These results confirm the prediction of the speciation theory that individual carcinomas have individual phenotypes, because they have individual karyotypes.

A common viral etiology would instead have predicted non-individual, common carcinoma-specific phenotypes shared by the seven virus-positive carcinomas listed above. Such virus-specific phenotypes would be lacking in the two virus-free carcinomas C-33A and HT-3. However, no such virus-specific phenotypes were observed.

To test whether the individual phenotypes of cervical carcinomas have karyotypic origins, we have next analyzed the karyotypes of the nine cervical carcinomas.

### Individual clonal karyotypes of carcinomas

**Figure 5:**
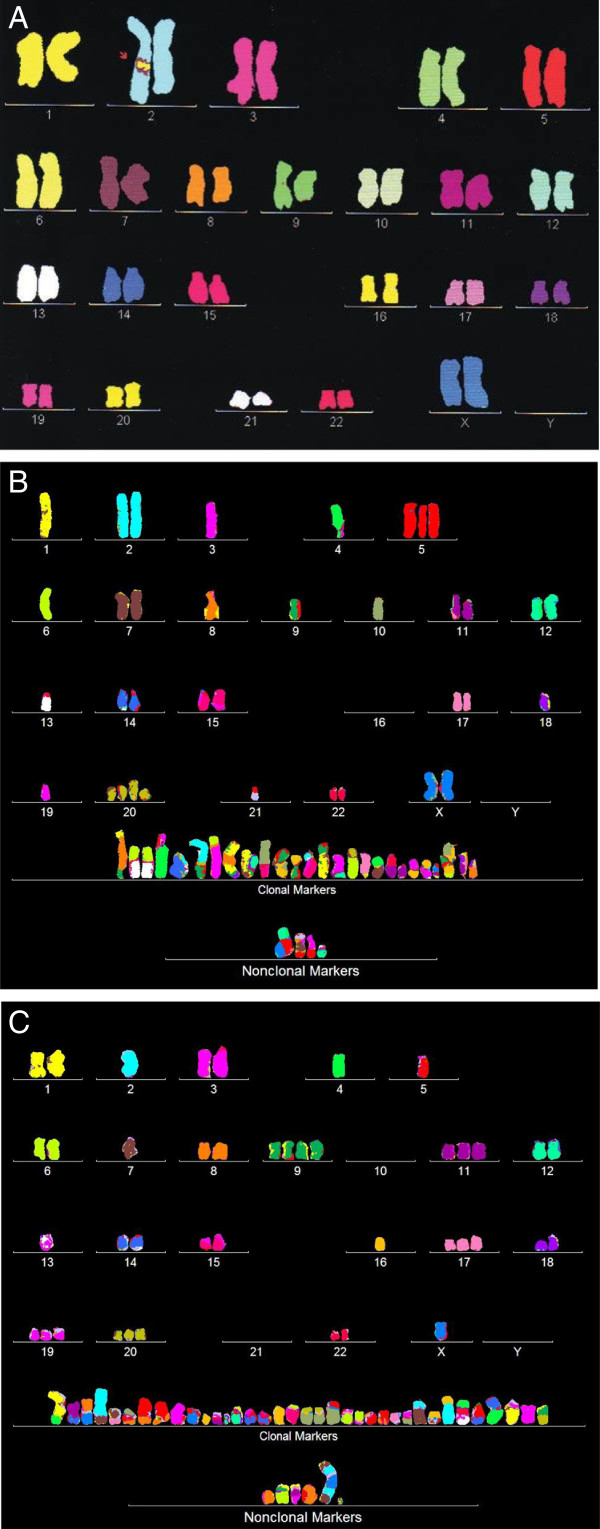
**Karyotypes of the normal human female (A) and the cervical carcinomas HT-3 (B) and CaSki (C).** The comparisons show that both carcinomas and the normal female had each distinct individual karyotypes. The karyotypes differed in their total chromosome numbers, the copy numbers of most intact chromosomes and in carcinoma-specific hybrid or marker chromosomes. The chromosomes were color-coded as described in Methods.

The speciation theory predicts that each cancer has an individual clonal karyotype. As a first test of the predicted karyotypic individuality we compared in Figure 5 the karyotypes of a normal female (Figure 5A) and of two cervical carcinomas, namely HT-3 (Figure 5B) and CaSki (Figure 5C). The comparisons show that both carcinomas differ much from each other and from the normal female karyotype in their total chromosome numbers, in the copy numbers of most intact chromosomes, and in the presence of carcinoma-specific hybrid or marker chromosomes. We will show next that the copy numbers of the intact and the marker chromosomes fall into a predominant clonal and into a minor non-clonal class.

**Figure 6:**
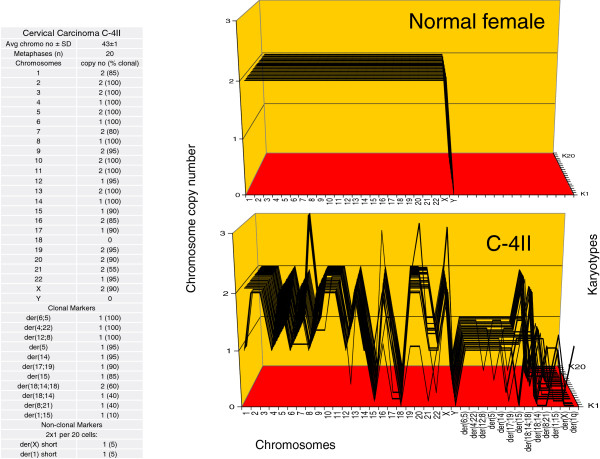
**Karyotype arrays of a normal female and of the near-diploid cervical carcinoma C-4II.** The figure shows the two karyotype arrays and a table indicating that the total chromosome number of C-4II is 43±1, and is thus quasi-clonal. The table further shows the %-clonality of all C-4II chromosomes. Karyotype-arrays are three-dimensional tables, which list the chromosome numbers of a given karyotype on the x-axis, the copy numbers of each chromosome on the y-axis, and the number of karyotypes arrayed on the z-axis. In such arrays the chromosomes of karyotypes with identical (clonal) copy numbers form parallel lines. The karyotype array with the attached table shows that 85 to 100% of the copy numbers of the C-4II chromosomes are clonal, and thus form parallel lines in the array. The minority of non-clonal C-4II chromosomes included (1) intact chromosomes with near-clonal copy numbers mostly oscillating in narrow margins of ±1 of clonal values and (2) two cells per 20 with unique non-clonal marker chromosomes. The presence of non-clonal intact and marker chromosomes indicates ongoing karyotypic variation predicted by the speciation theory (Background, Figure 1). By contrast, the karyotype array of a normal female shows 100%-clonality.

We have determined karyotypic clonality by comparing all chromosomes of 20 karyotypes of carcinomas in three-dimensional arrays [47,58]. These arrays are 3-dimensional tables, which list chromosome numbers on the x-axis, chromosome copy numbers on the y-axis and arrays of typically 20 karyotypes on the z-axis. Because all chromosomes of karyotypes with identical or clonal copy numbers form parallel lines in such arrays, it can be seen at a glance, whether and to what degree arrayed karyotypes are related. For example, the array of the 20 karyotypes of a normal human female, shown in Figure 6 (next paragraph), indicates at a glance that the copy numbers of the chromosomes of all 20 normal cells are two and thus clonal. Using such karyotype arrays we have investigated the clonalities of all nine cervical carcinomas studied here with the following results.

#### ***Individual clonal karyotypes of a normal female and of the near-diploid carcinomas C-4II and C-33A***

Figures 6 shows the karyotype arrays of a normal human female and of the near-diploid carcinoma C-4II together with a table listing the clonality of all C-4II chromosomes in %. As can be seen in the table, the average chromosome number of C-4II was 43±1 and thus near clonal (shown on the top of the table). The array and the table further show that the copy numbers of the C-4II chromosomes were 80 to 100% clonal and thus formed mostly parallel lines. The non-clonal chromosomes consisted (1) of intact chromosomes with non-clonal copy numbers mostly oscillating in narrow margins of ±1 of clonal values, and (2) of two unique marker chromosomes, which were present in 2 of 20 cells. By contrast, the 20 karyotypes of the normal female were 100% clonal. 

**Figure 7:**
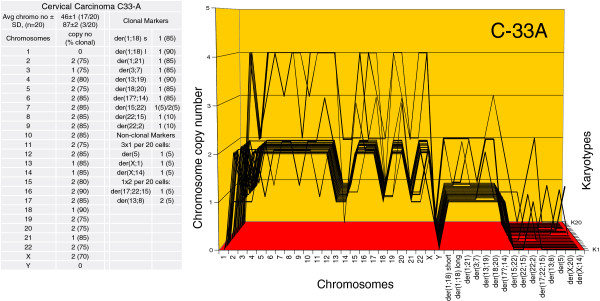
**Karyotype array of the near-diploid cervical carcinoma C-33A.** The C-33A array with the attached table, prepared as described in Figure 6, shows that the total chromosome number of C-33A is 46±1 and is thus quasi-clonal. The copy numbers of C-33A chromosomes were 70-90% clonal, forming parallel lines in the array. The non-clonal chromosomes included intact chromosomes with near-clonal copy numbers and new non-clonal marker chromosomes, which were found in four of 20 cells. A comparison of the arrays of C-33A and of C-4II shows at a glance that each carcinoma had an individual clonal karyotype.

Figure 7 shows that the average chromosome number of C-33A was 46±1 (see top of the attached table). The table with the karyotype array also shows that 70 to 90% of the copy numbers of the C-33A chromosomes were clonal, and thus formed parallel lines in the array. The non-clonal chromosomes consisted again of intact chromosomes with non-clonal copy numbers and of five unique marker chromosomes, which were present in 4 of 20 C-33A cells.

A comparison of the C-4II and C-33A arrays to each other (Figures 6 and 7) shows at a glance that the karyotypes of both carcinomas are highly clonal, and that they are individually very different, much like two different signatures. Moreover, the arrays of both carcinomas differ substantially from that of the normal female.

The presence of non-clonal chromosomes in both carcinomas indicates ongoing chromosomal variation despite overall clonality. In addition the C-33A displayed flexibility of the karyotype as a whole, namely with regard to its ploidy. An approximate two-fold polyploidization of the basic near-diploid karyotype was observed in 3 per 20 cells. Other examples of polyploidizations in cancers are listed as cancer-specific forms of karyotypic flexibility in Table 1, in figures below, in the Background and in the literature [21,46,50,55,58,89].

Thus the karyotype arrays of C-4II and C-33A confirm the individualities and clonalities of the karyotypes of carcinomas predicted by the speciation theory. Moreover, these karyotypes indicate a 1-to-1 ‘genotype-phenotype’ correspondence between individual clonal karyotypes and cellular phenotypes (Figure 2A,B) of the two carcinomas: ‘one carcinoma – one karyotype’.

In the following we have analyzed the karyotype arrays of the seven near-triploid cervical carcinomas introduced above (Table 1). According to the speciation theory the near-triploid carcinoma karyotypes should be more flexible and thus more heterogeneous than the near-diploid carcinomas, because more mitotic genes will be unbalanced in near-triploid than in near-diploid cancer karyotypes [60,62,90] (Background, Figure 1).

#### ***Individual clonal karyotypes of the near-triploid carcinomas SiHa, HT-, ME-180, CaSki, SW-756, HeLa and MS-751***

**Figure 8:**
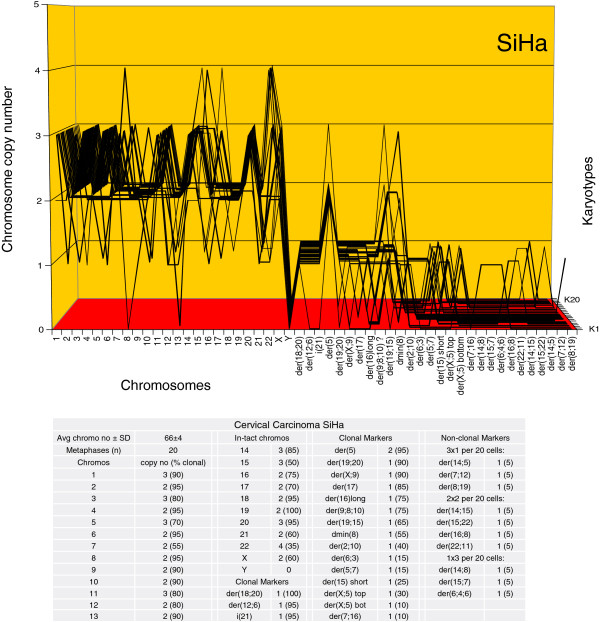
**Karyotype array of the near-triploid cervical carcinoma SiHa.** The SiHa array with the attached table, prepared as for Figure 6, shows that the total chromosome number is quasi-clonal at 66±4. The copy numbers of 60-100% of the chromosomes were clonal forming parallel lines in the array. The non-clonal chromosomes included intact chromosomes with near-clonal copy numbers and non-clonal marker chromosomes, which were found in six of 20 cells. Comparison of the karyotype array of SiHa with those of the near-diploid carcinomas C-4II and C-33A (Figures 6 and 7) shows at a glance that all three carcinomas have distinct, individual karyotypes.

**Figure 9:**
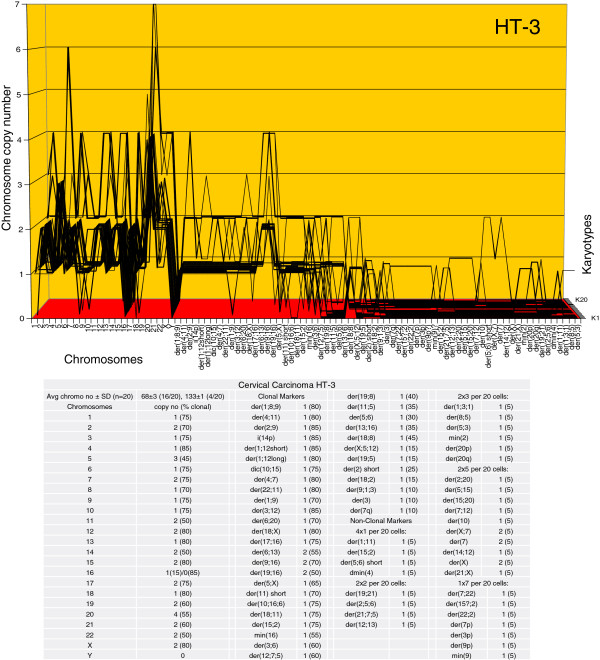
**Karyotype array of the cervical carcinoma HT-3.** The HT-3 array with the attached table shows that the total chromosome number is quasi-clonal at 68±3. The copy numbers of 60-85% of the chromosomes were clonal forming parallel lines in the array. The non-clonal chromosomes included intact chromosomes with near-clonal copy numbers and new non-clonal marker chromosomes, which were found in 14 out of 20 cells. Comparison of the karyotype array of HT-3 with those of the three carcinomas shown above in Figures 6, 7 and 8 reveals at a glance that all four carcinomas have distinct individual karyotypes.

**Figure 10:**
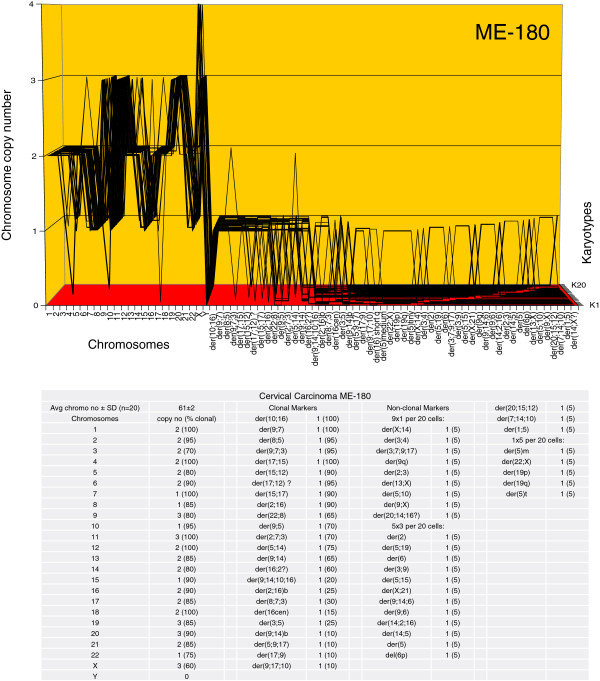
**Karyotype array of the cervical carcinoma ME-180.** The ME-180 array with the attached table shows that the total chromosome number is quasi-clonal at 63±2. The copy numbers of 60-100% of the chromosomes were clonal forming parallel lines in the array. The non-clonal chromosomes included intact chromosomes with near-clonal copy numbers and new non-clonal marker chromosomes found in 16 of 20 cells. Comparison of the karyotype array of ME-180 with those of the four carcinomas shown above in Figures 6, 7, 8 and 9 reveals again at a glance that all five carcinomas have distinct individual and clonal karyotypes.

**Figure 11:**
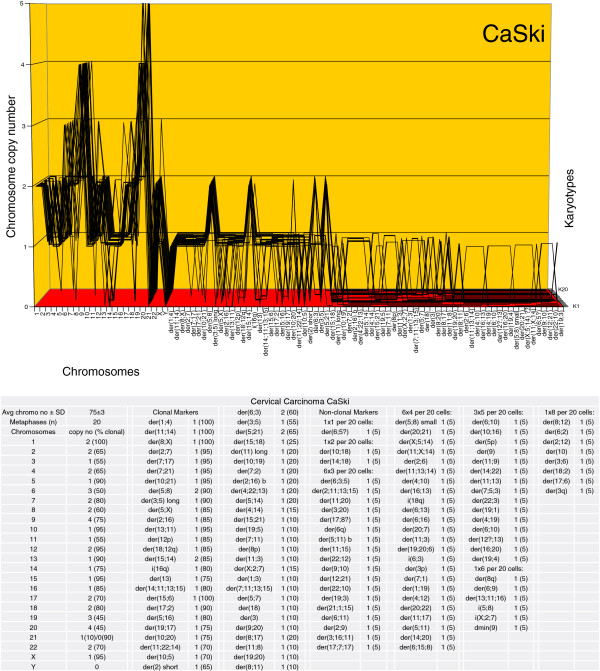
**Karyotype array of the cervical carcinoma CaSki.** The CaSki array with the attached table shows that the total chromosome number is quasi-clonal at 75±3. The copy numbers of 65-100% of the chromosomes were clonal forming parallel lines in the array. The non-clonal chromosomes included intact chromosomes with near-clonal copy numbers and new non-clonal marker chromosomes found in 19 of 20 cells. Comparison of the karyotype array of CaSki with five carcinomas shown above in Figures 6, 7, 8, 9 and 10 shows at a glance that all six carcinomas have distinct individual and clonal karyotypes. Note, about 60 non-clonal marker chromosomes of the 20 karyotypes of CaSki listed in the table are not shown in the graph to allow sufficient resolution for the clonal chromosomes.

**Figure 12:**
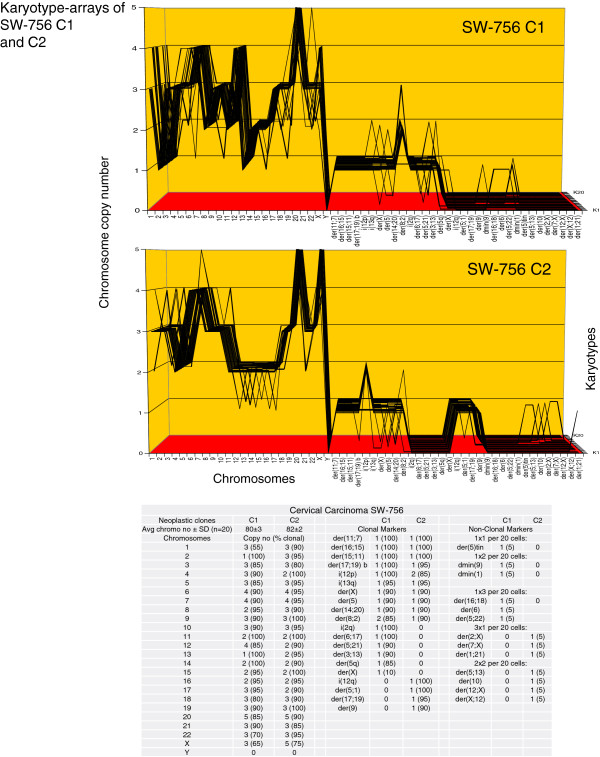
**Karyotype arrays of two morphological variants of the cervical carcinoma SW-756.** The table of chromosomes and the arrays of the SW-756 variants show that the total chromosome number of the round (C1) SW-756 clone is 80±3 and that of the fusiform (C2) clone is 82±2. Thus both clones are quasi-clonal. The table also shows that copy numbers of 55-100% of chromosomes of both clones formed parallel lines in the arrays. Non-clonal chromosomes included intact chromosomes with near-clonal copy numbers and new non-clonal marker chromosomes found in three and five of 20 SW-756 cells respectively. Comparison of the karyotype arrays of the two morphological SW-756 variants indicate that the two clones are phylogenetically closely related, but differed in the clonal copy numbers of seven intact chromosomes, and in five highly clonal round C1-specific and four fusiform C2-specific markers. Comparison of the SW-756 arrays with the arrays of the six carcinomas shown above in Figures 6, 7, 8, 9, 10 and 11 reveals again at a glance that all seven carcinomas have individual clonal karyotypes.

**Figure 13:**
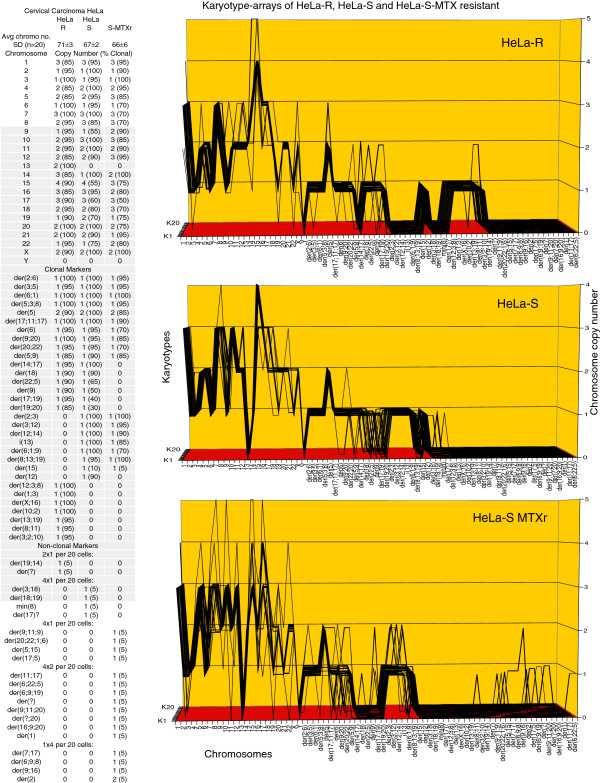
**Karyotype arrays of two morphological variants and a methotrexate-resistant variant of the cervical carcinoma HeLa.** The table of the chromosomes and the arrays of HeLa variants show that the total chromosome number of the morphological variant R (round) is 71±3, that of the morphological variant S (fusiform) is 67±2, and that of the methotrexate-resistant clone of S is 66±6. These chromosome numbers are thus all quasi-clonal. The copy numbers of 55-100% of chromosomes were also clonal. Non-clonal chromosomes included intact chromosomes with near-clonal copy numbers and new non-clonal marker chromosomes, which were found in two and four of the established morphological HeLa variants and in nine of 20 cells of the new methotrexate-resistant HeLa S clone. Comparisons of the karyotype arrays of the three HeLa variants show that all three variants are phylogenetically closely related subspecies with the following distinctions: The morphological variant R differed from S in five copy numbers of intact chromosomes and in 7 R-specific and 8 S-specific marker chromosomes. The methotrexate-resistant S clone differed from the native S variant in nine copy numbers of intact chromosomes and in the loss of seven 30-90% clonal S markers. Comparison of the HeLa arrays with the arrays of the seven distinct carcinomas shown in Figures 6, 7, 8, 9, 10, 11 and 12 reveals again at a glance that all eight carcinomas tested so far have individual and clonal karyotypes.

**Figure 14:**
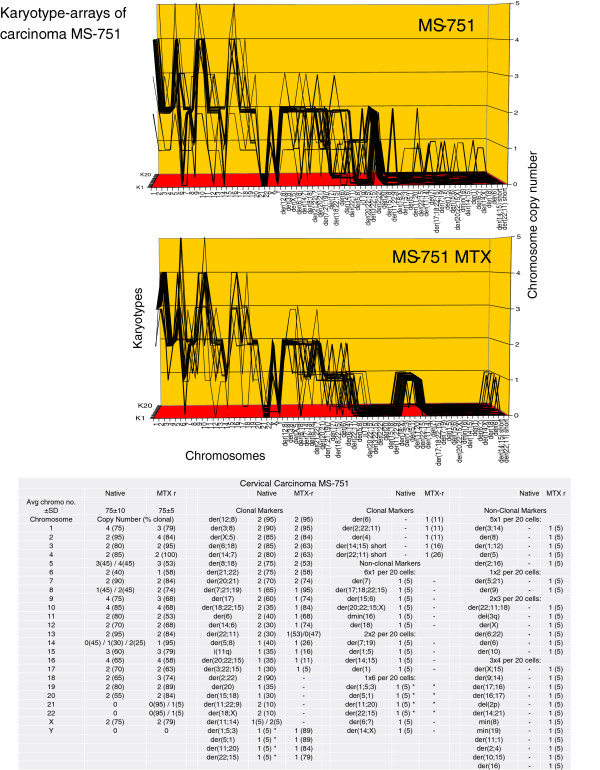
**Karyotype arrays of the native and a methotrexate-resistant variant of the near-triploid cervical carcinoma MS-751.** The table of the chromosome and the arrays of the two MS-751 variants show that the chromosome number of the native MS-751 variant is 75±10 and that of the methotrexate-resistant variant is 75±5. They are thus both quasi-clonal. The table also shows that the copy numbers of 60-100% chromosomes were clonal forming parallel lines in the karyotype arrays. Non-clonal chromosomes were intact chromosomes with near-clonal copy numbers and new non-clonal marker chromosomes, which were found in nine of 20 cells of the native MS-751 variant and in 11 of 20 cells of the drug-resistant clone. Comparison of the arrays of the two MS-751 variants shows at a glance that the two variants are karyotypically closely related. They differed from each other in the copy numbers of five clonal intact chromosomes, and in three 30–90%-clonal native MS-751-specific markers, and in four 80-90%-clonal methotrexate resistance-specific markers. By chance, the four clonal methotrexate-specific MS-751 marker chromosomes, flagged with asterisks, were all found in one non-clonal cell of the native MS-751 carcinoma (See also section three). Comparison of the MS-751 arrays with the arrays of the eight carcinomas shown in Figures 6, 7, 8, 9, 10, 11, 12 and 13 reveals again at a glance that all nine carcinomas have distinct individual clonal karyotypes. Note, about 25 non-clonal marker chromosomes of the 20 karyotypes of the MS-751 variants listed in the table are not shown in the graphs to allow sufficient resolution for the clonal chromosomes.

The karyotype arrays of the seven near-triploid cervical carcinomas SiHa, HT-3, ME-180, CaSki, SW-756, HeLa and MS-751 are shown in Figures 8, 9, 10, 11, 12, 13 and 14 respectively. As shown in the tables attached to each array, the total chromosome numbers of the near-triploid carcinomas were near-clonal with averages ranging from 63 to 81. The tables also show that the majority of the chromosomes of each carcinoma is highly clonal and thus formed predominantly parallel lines in the arrays shown in Figures 8, 9, 10, 11, 12, 13 and 14.

Thus far the karyotype arrays of nine cervical carcinomas illustrate at a glance the individuality and clonality of each carcinoma. Comparisons of the karyotypes of all carcinomas with their cellular phenotypes (Figures 2, 3 and 4) further indicate classical, individual ‘genotype-phenotype’ correlations [85]. Thus all cervical carcinomas studied here confirm the ‘one carcinoma-one karyotype’ rule shown to apply to the two near-diploid carcinomas C-4II and C-33A above [50].

As expected from the high degrees of aneuploidy (relative to normal cells) of the seven near-triploid carcinomas, the karyotypes of the near-triploid carcinomas (1) were more flexible and thus more heterogeneous, (2) included much higher numbers of clonal marker chromosomes, and (3) included higher percentages of cells with unique, non-clonal marker chromosomes than their near-diploid counterparts (see Background and next section).

#### ***Carcinoma-specific chromosome recombination rates***

In the following we have calculated individual “chromosome recombination indices” for each of the nine cervical carcinomas based on the percentages of their cells with unique, non-clonal marker chromosomes (Table 3). These percentages ranged from a low of 10% in near-diploid C-4II cells to a high of 95% in near-triploid CaSki cells. The dependence of these chromosome recombination rates on the degree of aneuploidy of the carcinoma karyotype is thus consistent with the prediction of the speciation theory that chromosomal instability is proportional to the degree of aneuploidy (Background).

**Table 3:** Carcinoma-specific chromosome recombination indices

**Cervical carcinoma**	**% cells with non-clonal markers**
C-4II	10
C-33A	20
SiHa	30
HT-3	70
ME-180	80
CaSki	95
SW-756 C1 and C2	15 and 25
HeLa R and S	10 and 20
MS-751	45

The relatively low chromosome recombination index of the near-triploid HeLa cell line shown in Table 3 may reflect evolutionary time-dependent selective balancing of mitosis genes in cancer species. The HeLa cell line was the first to be established in vitro, and has been stably maintained in many laboratories since 1952 [91].

*Section-specific conclusions*. 1) All (100%) of the carcinomas tested here (Table 1) contained individual, clonal karyotypes as predicted by the speciation theory (Figures 6, 7, 8, 9, 10, 11, 12, 13 and 14). The clonality of these carcinomas indicates that they derived from a single ancestral cell, in which this clonogenic karyotype was formed. Since these individual karyotypes correlated 1-to-1 with individual cell morphologies (Figures 2, 3 and 4), individual intrinsic drug-resistances (Table 2) and individual chromosome recombination indices (Table 3), and are not found in normal cells (see Figure 6), we conclude that these karyotypes generated and maintain the carcinomas studied here.

This conclusion agrees with our failure to find common and thus non-individual karyotypic or phenotypic characteristics shared by the seven virus-positive carcinomas. It also explains the failures of many others to find common virus-specific cytogenetic markers predicted by the virus-carcinoma theory [4,5,12,17-21,29,33,67].

2) The nine carcinoma karyotypes analyzed here also confirmed the individual karyotypic flexibilities predicted by the speciation theory (Background). Moreover the arrays shown in Figures 6, 7, 8, 9, 10, 11, 12, 13 and 14 and the carcinoma-specific chromosome recombination indices listed in Table 3 show that the karyotypic flexibilities and corresponding karyotypic heterogeneities are roughly proportional to the karyotypic complexity of carcinomas, as predicted by the speciation theory.

3) With regard to the relevance of carcinoma karyotypes to Pap-tests we deduce that karyotype analyses could provide new, DNA content-independent distinctions between carcinomas with near-normal DNA contents and low Pap-grades such as C-4II and C-33A and normal diploid hyperplastic tissues (Figures 6 and 7). Among the Pap-tests with high, near-triploid DNA contents, karyotype analyses could provide new information like the chromosome recombination index as a measure to grade malignancy.

### Do karyotypic variations or mutations alter the morphology and drug-sensitivity of carcinomas?

Since all nine individual carcinomas we analyzed contained individual clonal karyotypes and phenotypes, we deduced that individual karyotypes encode these carcinomas - just like the individual karyotypes that encode conventional species. This theory predicts that carcinomas are subject to phenotypic alteration by karyotypic variations, which would thus be sub-speciations. Previously, we have found that alterations in the sensitivity of cancers to toxic drugs [55,88], and the origins of metastases [50,57] correlated with karyotypic alterations.

By contrast, the viral and mutation theories of carcinomas postulate that poorly-defined viral sequences and gene mutations determine the phenotypes of carcinomas (Background).

To distinguish between the mutation and speciation theories as mechanisms of phenotypic variation, we compared here the karyotypes of two matched pairs of carcinomas differing in cell morphology, and of two matched pairs of carcinomas differing in acquired drug-resistance.

As can be seen in Figure 12 the karyotypes of the SW-756 clone with round cells (C1) differed from the karyotype of the clone with fusiform cells (C2) (Figure 4A,B) in seven clonal copy numbers of intact chromosomes and in six round clone C1-specific and four fusiform clone C2-specific marker chromosomes.

Likewise we show in Figure 13 that the karyotype of the HeLa R clone with round cells differed from the karyotype of the HeLa clone S with fusiform cells in the clonal copy numbers of five intact chromosomes and in six R-specific and eight S-specific marker chromosomes.

Similarly we show in Figure 13 that the karyotype of the methotrexate-resistant HeLa S clone (0.5 μg per 10^6 cells) differed from the karyotype of the drug-sensitive HeLa S clone in the copy numbers of 10 intact chromosomes and the loss of seven parental markers.

Likewise we show in Figure 14 that the karyotype of the methotrexate-resistant MS-751 clone differed from the karyotype of the drug-sensitive clone in the copy numbers of eight intact chromosomes, and the loss of one and the gain of 4 new clonal marker chromosomes.

By chance, we found that the four new methotrexate-specific MS-751 marker chromosomes were derived from one non-clonal cell of the native MS-751 carcinoma (Figure 14). To emphasize this accidental discovery, we flagged these markers in Figure 14 by an asterisk in both cases. Thus drug-resistance in this case was probably generated in part by the clonal selection of a non-clonal variant of the drug-sensitive native carcinoma MS-751. This confirms a previous example for phenotypic variation by selection of a non-clonal chromosome by Heng et al. [51,56].

*Section-specific conclusions.* 1) In sum carcinoma-specific phenotypes vary 1-to-1 with carcinoma-specific karyotypes. In view of this we conclude that karyotypic alterations or subspeciations cause alterations of the cell morphology and drug-sensitivity of carcinomas, rather than mutations or viral sequences. According to Marguerite Vogt in 1959, “a one-to-one relationship between karyotype and phenotype … favor(s) strongly the assumption of a causal relationship between karyotype and phenotype in a tissue culture line of human neoplastic cells, the strain HeLa” – much as we propose here now, 54 years later [85].

2) It could be argued, however, that the morphological variants of SW-756 and of HeLa, and the drug-resistant variants of HeLa and of MS-751 studied here were each caused by undetected mutations of cellular or viral genes, but were inadvertently accompanied by new clonal karyotypic variations. But it is unlikely that in all four of these variations undetected causal mutations were inadvertently accompanied by karyotypic alterations that were clonally stable in subsequent passages. It would follow that specific karyotypic variations, rather than mutations generated the morphological and drug-resistant carcinoma variants.

### Copy numbers of hundreds of over- and under-expressed mRNAs of individual carcinomas are proportional to the copy numbers of their chromosomes

Several researchers have recently investigated the transcriptomes of cervical carcinomas. As is the rule with other cancers (Background), the transcriptomes of cervical carcinomas consist of hundreds of individually over- and under-expressed normal mRNAs [33,45,68-72]. In agreement with the individuality of the transcriptomes of carcinomas, no consistent or “recurrent” carcinoma-specific “drivers” emerged in searches for common carcinoma-specific transcripts [33,45,69,70].

Moreover, it has not been possible to identify consistent transcriptomes of cervical carcinomas with HPV-functions [33,45], “likely representing common signaling pathways triggered by HPV” [68] and thought to have “potential value of targeting E6 and E7 function … in the treatment of HPV(+) cancers” [45]. This is again consistent with the absence of a common, virus-specific karyotypic pattern in the different virus-positive carcinomas described in section two above.

In view of this we propose here that the individual karyotypes of cervical carcinomas encode the complex, individual transcriptomes of carcinomas (Figure 1). This proposal is consistent with several independent studies, which have shown that the copy numbers of the mRNAs of cancers are directly proportional to the copy numbers of the corresponding chromosomes [71,73-75,77,78]. Accordingly Landry et al. have even shown by quantitative sequence analyses that the copy numbers of *all* HeLa mRNAs are exactly proportional to the copy numbers of the corresponding chromosomes. In view of this they concluded that there is “no dosage compensation” for the chromosome copy number-dependent dosage of HeLa mRNAs [71]. Adey et al. have just confirmed this conclusion [72].

*Section-specific conclusions*. We conclude that individual karyotypes of cervical carcinomas encode their highly complex, individual transcriptomes. In view of this we see the exact correlations between the copy numbers of the mRNAs and the copy numbers of the corresponding chromosomes of HeLa cells described by Landry et al. [71] and Adey et al. [72] as Rosetta stones of functional evidence for our theory.

### Additional karyotype-dependent characteristics of cervical carcinomas

All cancers have common characteristics in addition to those studied here, as for example (a) autonomy, (b) immortality and (c) the ability to metastasize [49,59]. Indeed all nine carcinomas studied here are autonomous and immortal in cell culture [84] and three of these, HT-3, MS-751 and CaSki have been described to form metastases in vivo [21].

The following reasons indicate that these cancer-specific commonalities are properties of common cancer-specific karyotypes rather than of common mutations or virus-derived sequences: (a) Autonomy is the primary characteristic of a species conferring reproductive independence of the parental species. As we pointed out above, the origin of carcinoma-specific autonomy coincides with that of the clonal origins of carcinoma-specific karyotypes (Background, Figure 1, Results, sections two and three). (b) Immortality of carcinomas has been reduced to karyotypic flexibility, which generates new variants constitutively to replace the inevitable chromosomal and genetic mutations that accumulate over time [58] (Figure 1). As described in the Background karyotypic flexibility and the corresponding cancer-specific clonal heterogeneity are direct consequences of the congenital aneuploidy of all cancers. (c) Metastasis has also been reduced to karyotypic flexibility generating metastatic sub-species of carcinomas with variant tissue-tropisms [50,57,59] – much as karyotypic flexibility generated the drug-resistant and morphological variants described above in Figures 12, 13 and 14.

*Section-specific conclusions*. Common carcinoma-specific characteristics like autonomy and karyotypic flexibility, which generate immortality and metastases, lend additional support to the theory that carcinoma-specific karyotypes are sufficient to create and maintain carcinomas, independent of mutations and sequences of latent viruses.

## Discussion

### 1) New individual karyotypes encode carcinomas - much like the individual karyotypes that encode conventional species

Since the individual clonal karyotypes of nine carcinomas correlated 1-to-1 and co-varied 1-to-1 with the complex individual transcriptomes and phenotypes of these carcinomas, we conclude that individual clonal karyotypes encode these carcinomas, much like the individual clonal karyotypes that encode conventional species. In sum this evidence provides genetic and functional proof for the speciation theory of carcinomas.

Indeed 1-to-1 correlations between phenotypes and karyotypes are the classical genetic basis of defining phylogenetic identities and relationships between conventional species [92,93]. Thus the endless individual “DNA contents” and corresponding individual karyotypes, grades of malignancy and histological phenotypes of the carcinomas studied here and of those first described by Papanicolaou et al. [1], each support the conclusion that carcinomas are species of their own.

Our conclusion that transcriptionally very active and complex individual karyotypes, instead of mutations or latent viral sequences, encode carcinomas thus explains our failures and those of numerous others to find common, virus-specific karyotypic or phenotypic characteristics in papilloma virus-positive carcinomas [5,12,16,18-21,29,33,67,70]. The carcinoma-species-theory also explains the recent finding that “HPV status … showed no correlation to outcome” [70].

Above all there is no direct functional evidence that cervical carcinomas depend on (1) the plethora of “passenger mutations” and “single nucleotide polymorphisms”, which are found in all cancers and normal cells by genome sequencing [94-96], or on (2) the defective and latent papilloma viral sequences (Background), which are found in 50 million or about 30% of healthy American females [37]. If carcinomas would depend on papilloma virus functions they would be immunogenic and subject to anti-viral immunity, which is not observed [31,34]. Indeed, the occurrence of papilloma virus-free cervical carcinomas is by itself sufficient evidence that cervical carcinomas are virus-independent (Background).

In view of this the clonal, but nonidentical viral sequences and cellular mutations found recently in individual cancers by whole genome sequencing appear to be non-carcinogenic, preneoplastic mutations, which became clonal accidentally by subsequent evolutions of clonogenic cancer-specific karyotypes.

Because of their lack of carcinogenic function such preneoplastic mutations have been termed “passenger mutations” [95-97] or “implausible” mutations [98]. Cancer cells are thought to acquire such individual passenger or implausible mutations during their long preneoplastic histories either from conception to cancer [95,96,99,100] or from virus infections 20–50 years prior to carcinogenesis [26]. Because of these long preneoplastic histories such mutations are also termed “archaeological” mutations [95,96].

In sum, our data indicate that newly formed carcinoma-specific karyotypes generate and maintain carcinomas, independent of latent viral sequences or mutations of tumor suppressor genes. Based on our findings it is expected that a vaccine against human papilloma viruses will have no effect on the occurrence of cervical carcinomas.

### 2) Karyotypic mechanism of carcinomagenesis

In searching for alternatives of the putative viral and mutational cervical carcinogens we found in the literature a long-known cervical carcinoma-specific carcinogen, namely cigarette smoke. Surprisingly, in view of the current viral and mutational theories, smoking was already known in the 1970s to induce cervical carcinomas [27,80-83]. In 1983 a study of the Centers for Disease Control even proved that “12 or more pack-years smoked” increase cervical carcinoma rates 12.4-fold compared to non-smokers [79]. The steady decline of cervical cancer in parallel with declining smoking rates in the US supports this theory [38]. Other studies have independently shown that smoking induces preneoplastic aneuploidy in proportion to the dose of cigarettes smoked [101-105]. In addition ionizing radiation has been found to induce cervical carcinomas too [106]. Thus, the induction of aneuploidy by cigarette smoke or other carcinogens supports the karyotypic mechanism of carcinomagenesis proposed here, and explains its characteristic time-dependence owing to the inevitably slow karyotype mutation-selection scheme outlined by the speciation theory (Background, Figure 1).

The “early” preneoplastic or even neoplastic karyotypic alterations of cervical biopsies described by several investigators lend unexpected support to the karyotypic mechanism of carcinomagenesis proposed by our theory [5,17,21,33,66,70,107,108] (see also previous section). These investigators may have used the proposition “early” for karyotypic alterations, because they expected early carcinoma causing mutations instead.

The speciation theory thus presents a coherent mechanism of cervical carcinomagenesis in which carcinogens initiate carcinogenesis by inducing aneuploidy, and aneuploidy then initiates automatic chain reactions of karyotypic evolutions that eventually generate new carcinoma-specific karyotypes.

## Conclusions

Since conventional DNA cytometry has a blind spot for near-diploid carcinomas and cancers, we propose that the karyotype analyses performed here could provide new, absolute distinctions between normal diploid but hyperplastic cervical tissues, hyperplasias with random aneuploidies and near-diploid carcinomas with clonal aneuploid DNA contents and low Pap-grades, such as C-4II and C-33A (Figures 6 and 7).

Moreover, our results promise new distinctions in grading malignancy of biopsies with high near-triploid DNA contents - based on the number of clonal versus non-clonal chromosomes, and on the corresponding chromosome recombination indices described here (Table 3). This speculation seems justified, because our results confirm the rule (Background): The more “abnormal” the “DNA content” – the higher is the flexibility and thus the malignancy of the carcinoma [1,3-6,10,17,59,65,67].

Thus the karyotypic mechanism of carcinomagenesis described here offers new, testable karyotypic markers to distinguish between benign diploid biopsies, as yet benign biopsies with non-clonal preneoplastic aneuploidies [58], and malignant carcinomas with clonal near-diploid and near-triploid karyotypes like those shown in Figures 6, 7, 8, 9, 10, 11, 12, 13 and 14.

In view of this we think our results provide the basis for a new and improved approach to the diagnosis, treatment and prevention of cervical carcinomas along the lines of the original discoveries of Papanicolaou et al. [1].

## Methods

### Cells and cell culture

The papilloma virus-free human cervical carcinoma cell lines C-33A and HT-3, and the viral DNA-positive lines C-4II and CaSki were obtained from American Type Culture Collection (Manassas, VA). The cervical carcinoma lines SiHa, ME-180, MS-751, SW-756 and a second strain of CaSki were kind gifts of Pulivarthi Rao (Laboratory of Molecular Cytogenetics, Texas Children's Cancer Center, Baylor College of Medicine, Houston, Texas). The HeLa line was a kind gift of Ann Fischer (Cell culture facility at UC Berkeley, Barker Hall). Cells were propagated in cell culture medium RPMI 1640 (Sigma Co) supplemented with 5% or 10% fetal calf serum, 1% Anti-Anti (GibCo company) and 1% Nystatin (Sigma company).

### Karyotype analysis of cervical carcinoma cells

One to two days before karyotyping, cells were seeded at about 50% confluence in 3 ml medium containing 5% fetal calf serum in a 5-cm culture dish. After reaching ~75% confluence, 200 ng colcemid in 20 μl solution (KaryoMax, Gibco Invitrogen company) was added to 3 ml medium. The culture was then incubated at 37°C for 4–8 hrs. Subsequently cells were dissociated with trypsin, washed once in 3 ml of physiological saline, and then incubated in 0.075 molar KCl at 37°C for 15 min. After cooling the solution in ice-water, 0.1 volume of freshly mixed glacial acetic acid-methanol (1:3, vol. per vol.) was added and the mixture was centrifuged at 800 g for 6 minutes at room temperature. The cell pellet was then suspended drop-wise in ice-cold acetic acid-methanol (1:3) solution and incubated at room temperature for 15 min. This cell suspension was then pelleted by centrifugation as described above, resuspended in an appropriate small volume and either transferred onto a microscope slide with a micropipette tip to give a suitable density of metaphases for microscopic analysis or stored at -20C. Slides with suitable metaphase chromosomes were hybridized with chromosome-specific color-coded DNA probes (MetaSystems, Newton, MA 02458), as described by the manufacturer and by us previously [45,83], to generate the chromosome-specific colors shown above in Figure 5.

## Note added in proof

Stepanenko and Kavsan have recently published an independent speciation theory of cancer similar to the one described here [109].

## Competing interests

The authors declare that they have no competing interests.

## Authors’ contributions

AM, JF, MD, MB and PD carried out experimental work, karyotype analyses and participated in the preparation of the manuscript. CF participated in the preparation of the ms and the underlying concepts based on his medical practice. PD carried out experiments and wrote most of the manuscript with active cooperation by all authors. All authors read and approved the final manuscript.
